# Adiponectin and Leptin Trajectories in Mexican-American Children from Birth to 9 Years of Age

**DOI:** 10.1371/journal.pone.0077964

**Published:** 2013-10-30

**Authors:** Vitaly Volberg, Brianna Heggeseth, Kim Harley, Karen Huen, Paul Yousefi, Veronica Davé, Kristin Tyler, Michelle Vedar, Brenda Eskenazi, Nina Holland

**Affiliations:** 1 Center for Environmental Research and Children’s Health (CERCH), School of Public Health, University of California, Berkeley, California, United States of America; 2 Department of Statistics, University of California, Berkeley, California, United States of America; John Hopkins bloomerg school of public health, United States of America

## Abstract

**Objectives:**

To address molecular mechanisms underlying obesity development, we examined patterns of critical metabolism-related hormones, adiponectin and leptin (adipokines), over childhood.

**Subjects and Design:**

Plasma adiponectin and leptin were measured in 80 Mexican-American children at birth and again at 2, 5, and 9 years from the ongoing prospective cohort followed by the Center for the Health Assessment of Mothers and Children of Salinas (CHAMACOS). We used a mixture modeling approach to identify patterns in adipokine trajectories from birth to 9 years.

**Results:**

Leptin was positively related to child body size within all ages, however adiponectin had inverse and weaker associations with BMI at 2, 5, and 9 years. Correlations between adipokine levels over the 0–2, 2–5, and 5–9-year periods increased for both leptin (r = 0.06, 0.31 and 0.62) and adiponectin (r = 0.25, 0.41 and 0.46). Our mixture modeling approach identified three trajectory clusters for both leptin (1L [slowly-rising], 2L [rapidly-rising], and 3L [stable]) and adiponectin (1A [steep-dropping and rebounding], 2A [moderately-dropping], and 3A [stable]). While leptin groups were most separated over the 2–9-year period, adiponectin trajectories displayed greatest heterogeneity from birth to 2 years. Children in the rapidly-rising 2L group had highest BMI and waist circumference at 9 years. Further, children with greater birth weight had increased odds of belonging to this high risk group (OR = 1.21 95% CI 1.03, 1.43, compared to stable group 3L). Children whose mothers consumed more sugar-sweetened beverages during pregnancy were at risk of being in the steep-dropping 1A group (OR = 1.08, 95% CI 1.01, 1.17, compared to stable group 3A).

**Conclusion:**

Our results highlight developmental differences in leptin and adiponectin over the childhood period. Leptin closely reflects child body size however factors affecting adiponectin and long-term consequences of its changes over infancy need to be further explored.

## Introduction

Recent data show that children may follow different growth patterns and that those who display excessively rapid body mass index (BMI) gains may be at higher risk for adult obesity and adverse metabolic health [1]–[5]. However, longitudinal data demonstrating changes in critical energy balance hormones, such as adiponectin and leptin (commonly termed adipokines), are limited. Examining adipokine trajectories over childhood may help shed further light on obesity etiology and heterogeneity of growth patterns.

Adiponectin, a protein hormone secreted by adipose tissue, targets muscle and liver to increase uptake and catabolism of fatty acids and carbohydrates, promoting insulin sensitivity [6]. In children, lower adiponectin levels are associated with both metabolic syndrome and type 2 diabetes [7], [8]. Further, while there is a well-established inverse relationship between adiponectin and BMI in school-age children, this relationship is less clear in newborns. Several studies report a positive relationship between adiponectin and birth weight but others do not [9]–[12].

Leptin, a hormone synthesized primarily by adipose tissue but also by the stomach, skeletal muscle and liver, acts on the hypothalamus to convey satiety and regulate long-term energy balance [13], [14]. Plasma leptin levels correlate positively with adiposity in newborns, children and adults [15], [16]. However, obese individuals commonly develop ‘leptin resistance’ or tolerance – a hyperleptinemic state characterized by decreased responsiveness to leptin [15].

An important and yet relatively unaddressed question is whether early life adipokine measures predict future adipokine levels and obesity-related outcomes. Only two prospective cohorts have explicitly examined such associations, both over a relatively narrow age range [17], [18]. Data from Project Viva (N = 588) showed that cord blood adiponectin was positively related to central obesity (p = 0.04) but not with adiponectin at 3 years [17]. Cord blood leptin was negatively related to BMI (p<0.001) and positively related to leptin (p = 0.001) at 3 years. Additionally, a report on older children (N = 519) showed that adiponectin and leptin in 9 to 10-year-olds were directly and significantly correlated with their respective adipokine levels, three years later (p<0.001 for both) [18]. While there is some evidence that early and later life adipokine levels are associated, it is unclear whether such relationships hold over the childhood period.

Emerging data show that the perinatal environment may influence adipokine trajectories. We have previously shown that greater rate of weight and length gain in the first 6 months of life is negatively associated with 9-year adiponectin levels, adjusting for concurrent child BMI [19]. Further, maternal nutrition, gestational diabetes, weight gain during pregnancy and child birth weight have been previously identified as risk factors for obesity and adverse metabolic health in later life, but their relationship with child adipokines remains poorly understood [1]–[3], [20]–[22].

To address current data gaps on adipokine development over childhood, we measured adiponectin and leptin levels in a prospective cohort of children at birth, 2, 5, and 9 years. We determined their associations between time points and with child BMI at each age. Further, using a mixture modeling approach, we identified groups of children with distinct adipokine trajectories and examined whether demographic, fetal growth and dietary factors are associated with these groups. Analyses were performed using data from the Center for the Health Assessment of Mothers and Children of Salinas (CHAMACOS), a Mexican-American cohort with a high prevalence of child obesity [23],[24].

## Materials and Methods

### Subjects and Study Design

The CHAMACOS study is a longitudinal birth cohort designed to assess the health effects of pesticides and other exposures on growth and development in children living in Salinas Valley, CA [25], [26]. Mothers were enrolled during pregnancy between October 1999 and October 2000, with 537 mother-child pairs in the study at delivery and 327 pairs remaining at the 9-year visit. Eligible women were ≥18 years of age, <20 weeks gestation at enrollment, English or Spanish speaking, Medi-Cal eligible and planning to deliver at the county hospital. Women were interviewed at ∼13 weeks gestation, ∼26 weeks gestation, shortly after delivery, and when their children were 6 months, and 1, 2, 3½, 5, 7, and 9 years of age. Developmental assessments of children, including anthropometrics, were conducted at birth and at the time of each maternal interview. Adiponectin and leptin were measured on a convenience sample of 80 children having blood samples at birth, 2, 5, and 9 years and complete anthropometric and demographic data. No differences were observed comparing maternal and child demographic and anthropometric measures between this sub-sample and the overall CHAMACOS cohort.

### Ethics Statement

We obtained signed informed consent from all women at the time of enrollment in the study. All study procedures were approved by the Committee for the Protection of Human Subjects at the University of California, Berkeley. IRB Protocol 2010-03-949.

### Questionnaire Data

Sociodemographic information, including maternal age at pregnancy, years of living in US prior to pregnancy, education, and household poverty category was gathered during the first prenatal interview at 14±5 weeks gestation. Interviews were conducted in Spanish or English by bilingual, bicultural trained interviewers. Maternal pre-pregnancy BMI was calculated using the mother’s self-reported pre-pregnancy weight and measured height. During the second prenatal interview at 26±3 weeks gestation we used a previously validated food frequency questionnaire (FFQ) to assess maternal sugar-sweetened beverage consumption [27], [28]. In brief, participant mothers were asked to report number of drinks in the last 3 months of 100% orange, grapefruit, apple, grape, or other real 100% fruit juice; fruit drinks (Tampico, Sunny D, lemonade, Kool-Aid); or non-diet soda (recoded as drinks per week). This FFQ is based on the Spanish-language Block 98 Questionnaire and was modified for use in the CHAMACOS Mexican-American population [27]. Additional details on the FFQ used here are available in Harley et al [28]. Data on maternal weight at delivery, infant birth weight, length, and gestational age were obtained from delivery medical records abstracted by a registered nurse. Children were categorized as ‘small for gestational age’ (SGA) if their birth weight was <10^th^ percentile for gestational age, controlling for ethnicity, parity and infant sex from national data [29]. They were categorized as ‘large for gestational age’ (LGA) if their birth weight was >90^th^ percentile. Children were considered as ‘at term’ if they were born at or after 37 weeks of gestation. Gestational weight gain was calculated as mother’s weight at delivery from medical records minus self-reported pre-pregnancy weight. Associations between maternal smoking and adipokines were not examined as 76 of the 80 mothers reported no use of tobacco at the prenatal visits.

### Anthropometric Measurements

Children’ weight and height were measured at the 2, 5, and 9-year visits using a calibrated electronic scale (Tanita Mother-Baby Scale Model 1582, Tanita Corp.) and stadiometer, respectively. Child height was measured in triplicate and the average of measurements was used. Child waist circumference was measured at the 9-year-visit with a tape placed above the crest of the ileum. Measurements were recorded to the nearest 0.1 cm after the child exhaled, performed in triplicate and the average of the measures was used. BMI was calculated as mass in kilograms divided by height in meters squared. Children were categorized as normal weight, overweight or obese using the sex and age-specific BMI cut-offs (85^th^ and 95^th^ percentile, respectively) provided by the 2000 Centers for Disease Control and Prevention (CDC) child growth data. Mothers were categorized as normal weight (18.5–24.9 kg/m^2^), overweight (25–29.9 kg/m^2^) or obese (≥30 kg/m^2^) using the standard CDC BMI cutoffs for adults.

### Plasma Adiponectin and Leptin Measurements

Adiponectin and leptin were measured in banked blood plasma samples stored at −80°C using enzyme-linked immunoassay (ELISA) RayBio Human Adiponectin and Human Leptin kits (Norcross, GA). The minimum detectable concentrations for adiponectin and leptin ELISAs were 10 pg/ml and 6 pg/ml respectively. All samples were run in duplicate and the values were averaged. The intra- and inter-plate coefficients of variance (CV) were 3% and 12%, respectively, for adiponectin and 3% and 13%, respectively, for leptin.

### Statistical Analyses

Adiponectin levels were approximately normally distributed, but leptin levels were right-skewed at all four time points. Thus, all leptin-based analyses use base-ten log-transformed values. We used Pearson’s correlation coefficient and linear regression to determine both associations between adipokines measured at different time points and associations between adipokine levels and child anthropometry.

To explore whether there are distinct adipokine trajectory groups, we used vertically-shifted mixture modeling (VSMM). This method is an extension to Gaussian mixture modeling, which is the basis of group-based trajectory modeling (GBTM) and growth mixture models (GMM) and focuses on clustering trajectories based on their shape over time [30]–[32]. A detailed description of this approach is provided in Heggeseth et al [33]. In brief, this method removes individual level means and assumes that within each group, 1) mean adipokine trajectories follow a piecewise polynomial function of time based on a 3^rd^ order B-spline with one knot at the median time, and 2) an exponential working variance-covariance matrix for the repeated adipokine measurements within participants. Overall, this approach seeks to identify mean patterns of adipokine trajectories and assigns individuals to these groups based on highest posterior probabilities using the expectation-maximization (EM) algorithm.

Best fit was assessed comparing the Bayesian information criterion (BIC) for 1 to 5 group models. Group membership was simultaneously modeled using a generalized logit function to estimate odds ratios (ORs) of group membership as a function of early life variables. Pearson’s correlation and linear regression analyses were conducted using STATA 12 (College Station, TX) for Windows. All mixture modeling analyses were performed using R statistical software (R Foundation for Statistical Computing, Vienna, Austria). P-values <0.05 were considered statistically significant.

## Results

### Maternal and Child Characteristics

Of the 80 children in this study, there were a similar number of boys (N = 39) and girls (N = 41) (Table 1). Children were delivered primarily at term (≥37 weeks, 95%) and were appropriate for gestational age (88%). Very few children were small for gestational age (<10^th^ percentile, 6%) or large for gestational age (>90^th^ percentile, 6%). At 9 years, the majority of children were overweight or obese (54%). At time of pregnancy, mothers tended to be young (25.6 years), have resided in the US 10 years or less (75%) and have low educational attainment (41% with ≤6^th^ grade). Additionally, more than half of the families (56%) were living at or below the federal threshold for poverty. Based on pre-pregnancy BMI, 55% of mothers were overweight or obese.

**Table 1:** Demographic characteristics of mothers and children from the CHAMACOS Study, Salinas Valley, CA (N = 80).

Characteristic	N (%)
**Child sex**	
Boy	39 (49)
Girl	41 (51)
**Child gestational age at birth**	
34–36 Weeks	4 (5)
≥37 Weeks	76 (95)
**Child birth size**	
Small for gestational age (<10th percentile)	5 (6)
Appropriate for gestational age	70 (88)
Large for gestational age (>90th percentile)	5 (6)
**Child BMI** 1 **at 9 years**	
Normal (≤85th percentile)	37 (46)
Overweight (>85th, <95th percentile)	17 (21)
Obese (≥95th percentile)	26 (33)
**Maternal age at pregnancy**	
18–24	39 (49)
25–29	22 (27)
30–34	15 (19)
35–45	4 (50
**Maternal years in US at pregnancy**	
<1	20 (25)
1–10	40 (50)
>10	20 (25)
**Maternal education at pregnancy**	
≤6th Grade	33 (41)
7–12 Grade	34 (43)
≥High School	13 (16)
**Household poverty category at pregnancy**	
≤Poverty threshold	45 (56)
>Poverty level but <200% poverty level	32 (40)
≥200% Poverty level	3 (4)
**Maternal pre-pregnancy BMI**	
Normal (18.5–24.9 kg/m^2^)	36 (45)
Overweight (25–29.9 kg/m^2^)	28 (35)
Obese (≥30 kg/m^2^)	16 (20)

1 Child’s weight status was determined using age and sex adjusted body mass index cut offs for 85th and 95th percentiles from CDC child growth charts.

### Child Adipokines by Age and Gender

Children had highest levels of adiponectin at birth (mean±SD, 112.8±35.1 µg/ml), with a subsequent decrease to lower values at 2 years of age (51.3±20.4 µg/ml) (Table S1). Adiponectin levels continued to decrease, however not as sharply, until 5 years (41.9±18.5 µg/ml), and remained similar at 9-years (42.5±19.6 µg/ml). Adiponectin did not differ between boys and girls at any of the time points assessed. Child log(leptin) decreased from initially high levels at birth (1.08±0.37) to lower levels at 2 years of age (0.42±0.16). At the following time points, there was a small but significant increase to 5-year levels (0.49±0.23), and a sharper increase to higher leptin in 9-year-olds (0.89±0.47). Girls had higher levels of leptin compared to boys at birth and at 5 years (p<0.001, p = 0.01, respectively), but only the difference at birth persisted after adjustment for child’s birth weight.

### Relationships between Child Adipokines and Size (Birth Weight and BMI) by Age

While child adiponectin levels at birth and birth weight were unrelated (β = 0.01, p = 0.47; r = 0.08), there was a progressively significant negative association between adiponectin and BMI at 2 (β = −2.91, p = 0.03; r = −0.24), 5 (β = −2.33, p = 0.01; r = −0.28), and 9 years (β = −2.06, p<0.001; r = −0.42) (Table 2). Leptin levels at birth were associated with birth weight (β = 0.001, p<0.001; r = 0.46) and the leptin – BMI relationship strengthened over the 2 (β = 0.05, p<0.001; r = 0.52), 5 (β = 0.07, p<0.001; r = 0.73), and 9-year (β = 0.09, p<0.001; r = 0.78) time points.

**Table 2:** Relationship between adiponectin, leptin, and size (birth weight and BMI) in children from birth to 9 years of age (N = 80).

	Beta^1^ (p)	r^2^
**Birth Weight - A_0_**	0.01 (0.47)	0.08
**BMI_2_ - A_2_**	−2.91 (0.03)	−0.24
**BMI_5_ - A_5_**	−2.33 (0.01)	−0.28
**BMI_9_ - A_9_**	−2.06 (<0.001)	−0.42
**Birth Weight - L_0_**	0.001 (<0.001)	0.46
**BMI_2_ - L_2_**	0.05 (<0.001)	0.52
**BMI_5_ - L_5_**	0.07 (<0.001)	0.73
**BMI_9_ - L_9_**	0.09 (<0.001)	0.78

A - adiponectin, L - log_10_(leptin). BMI - body mass index (kg/m^2^).

A_0_, L_0_ - at birth, A_2_, L_2_ - at 2 years, A_5_, L_5_ - at 5 years, A_9_, L_9_ - at 9 years.

1 Linear regression coefficient.

2 Pearson’s correlation coefficient.

### Associations between Adipokine Levels at Different Ages

Crude bivariate analyses showed that while adiponectin levels at birth were significantly but weakly associated with adiponectin at older ages, stronger correlations were observed over the 2–5 and 5–9-year periods (Table 3). Additionally, strength of correlations grew comparing birth – 2-years (β = 0.14, p = 0.03; r = 0.25), 2–5-years (β = 0.38, p<0.001; r = 0.41), and 5–9-years (β = 0.49, p<0.001; r = 0.46). Leptin at birth did not significantly predict later life leptin levels at either 2, 5, or 9 years. Leptin at 2 years was weakly but significantly associated with leptin at both 5 (β = 0.45, p = 0.004; r = 0.31) and 9 (β = 0.63, p = 0.05; r = 0.22) years. The strongest correlation was found between 5 and 9-year-old leptin levels (β = 1.26, p<0.001; r = 0.62).

**Table 3:** Associations within adipokines in children over the birth - 9-year period (N = 80).

	A_2_		A_5_		A_9_	
	Beta^1^ (p)	r^2^	Beta (p)	r	Beta (p)	r
**A_0_**	0.14 (0.03)	0.25	0.17 (0.003)	0.33	0.12 (0.05)	0.22
**A_2_**			0.38 (<0.001)	0.41	0.46 (<0.001)	0.48
**A_5_**					0.49 (<0.001)	0.46
	**L_2_**		**L_5_**		**L_9_**	
	**Beta (p)**	**r**	**Beta (p)**	**r**	**Beta (p)**	**r**
						
**L_0_**	0.03 (0.58)	0.06	0.12 (0.08)	0.20	0.24 (0.09)	0.19
**L_2_**			0.45 (0.004)	0.31	0.63 (0.05)	0.22
**L_5_**					1.26 (<0.001)	0.62

A - adiponectin, L - log_10_(leptin).

A_0_, L_0_ - at birth, A_2_, L_2_ - at 2 years, A_5_, L_5_ - at 5 years, A_9_, L_9_ - at 9 years.

1 Linear regression coefficient.

2 Pearson’s correlation coefficient.

### Description of Adipokine Trajectory Groups

**Figure 1:**
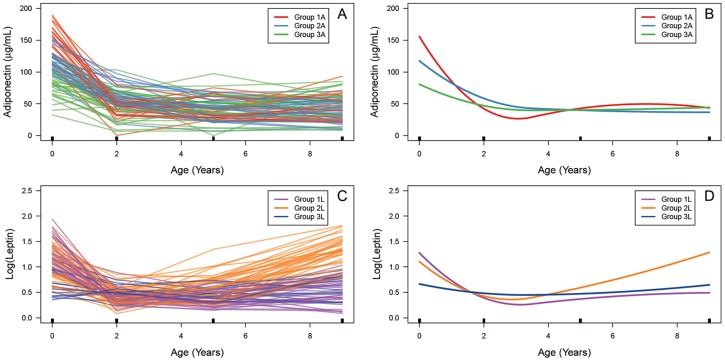
(A) Individual child adiponectin trajectories. Black rectangles indicate age at which adiponectin was measured. Orange, blue, and green trajectories comprise groups 1A, 2A, and 3A, respectively, as identified by vertically-shifted mixture modeling (VSMM). (B) VSMM-defined adiponectin trajectory groups. (C) Individual child leptin trajectories. Purple, orange, and dark blue trajectories comprise groups 1L, 2L, and 3L. (D) VSMM-defined leptin trajectory groups.

Mixture modeling analysis identified three distinct trajectory patterns for both adiponectin and leptin development over the birth – 9-year period (Figure 1). Adiponectin and leptin levels for these groups across birth, 2, 5, and 9 years are described in Table 4. P-values for differences across groups were not generated as mixture modeling approaches estimate clustering through use of posterior probabilities, assuming some amount of uncertainty in group assignment. For adiponectin, groups were most separated over the birth – 2-year period, with group 1A (N = 19) having the steepest decline in levels, group 2A (N = 31) showing a more moderate decline and group 3A (N = 30) showing the slowest decline. At birth, mean±SD adiponectin levels for 1A, 2A, and 3A were 155.7±22.7 µg/ml, 117.5±17.3 µg/ml and 80.8±21.0 µg/ml, respectively. These differences did not persist into older age, with groups 1A, 2A, and 3A settling at a similar level over the 2–9-year period. At 9 years, mean adiponectin levels for 1A, 2A, and 3A were relatively close, at 44.2±20.6 µg/ml, 37.5±15.8 µg/ml and 46.7±21.9 µg/ml, respectively. BMI at 2, 5, and 9 years and waist circumference at 9 years were similar for 1A, 2A, and 3A groups.

**Table 4:** Child adipokine levels and size by cluster.

	Adiponectin Group		
	1A (N = 19)	2A (N = 31)	3A (N = 30)
	mean (SD)	mean (SD)	mean (SD)
**A_0_ (µg/mL)**	155.7 (22.7)	117.5 (17.3)	80.8 (21.0)
**A_2_ (µg/mL)**	42.4 (16.8)	59.2 (17.5)	48.8 (22.8)
**A_5_ (µg/mL)**	43.1 (17.5)	40.5 (15.9)	42.7 (21.8)
**A_9_ (µg/mL)**	44.2 (20.6)	37.5 (15.8)	46.7 (21.9)
**Birth Weight (g)**	3496 (537)	3447 (364)	3425 (455)
**BMI_2_ (kg/m^2^)**	17.6 (1.3)	17.0 (1.9)	17.2 (1.7)
**BMI_5_ (kg/m^2^)**	17.1 (1.5)	17.5 (2.9)	17.0 (1.9)
**BMI_9_ (kg/m^2^)**	20.4 (3.6)	20.9 (4.3)	19.7 (4.1)
**Waist_9_ (cm)**	73.4 (10.0)	74.8 (12.1)	71.8 (11.5)
	**Leptin Group**		
	**1L (N = 27)**	**2L (N = 37)**	**3L (N = 16)**
	**mean (SD)**	**mean (SD)**	**mean (SD)**
**L_0_**	1.28 (0.28)	1.11 (0.34)	0.67 (0.24)
**L_2_**	0.39 (0.15)	0.43 (0.17)	0.48 (0.14)
**L_5_**	0.36 (0.14)	0.59 (0.26)	0.48 (0.13)
**L_9_**	0.48 (0.26)	1.29 (0.29)	0.63 (0.22)
**Birth Weight (g)**	3494 (428)	3518 (432)	3219 (422)
**BMI_2_ (kg/m^2^)**	16.5 (1.3)	17.6 (1.9)	17.5 (1.6)
**BMI_5_ (kg/m^2^)**	16.0 (1.1)	18.4 (2.6)	16.6 (1.6)
**BMI_9_ (kg/m^2^)**	17.3 (1.9)	23.3 (3.7)	18.5 (2.5)
**Waist_9_ (cm)**	64.9 (5.4)	81.4 (9.6)	68.9 (9.7)

A - adiponectin (µg/ml), L - log_10_(leptin).

BMI - body mass index. Waist - waist circumference.

A_0_, L_0_ - at birth, A_2_, L_2_ - at 2 years, A_5_, L_5_ - at 5 years, A_9_, L_9_ - at 9 years.

BMI_9_, Waist_9_ - at 9 years.

For leptin, groups 1L (N = 27) and 2L (N = 37) had similar rates of decline from birth to 2 years. However, from 2 to 9 years, group 1L grew slowly while group 2L showed a rapid increase in leptin levels. Group 3L (N = 16) showed minimal changes in leptin levels over the birth – 9-year period. Mean±SD log(leptin) at birth for 1L, 2L, and 3L was 1.28±0.28, 1.11±0.34 and 0.67±0.24, respectively. At 9 years, leptin levels had decreased to 0.48±0.26 for 1L, increased to 1.29±0.29 for 2L, and stayed relatively constant at 0.63±0.22 for 3L. Compared to 1L and 3L, birth weight, 2, 5, and 9-year BMI, along with 9-year waist circumference, were all highest for the rapidly-rising 2L group. We found only limited overlap in group membership comparing adiponectin to leptin groups.

### Associations between Early Life Factors and Adipokine Groups

Our analysis examined whether candidate perinatal factors may be associated with adipokine group membership, including sociodemographics at pregnancy (maternal age, years in US, education and family poverty category), child sex, gestational age, birth weight, birth length, maternal pre-pregnancy BMI, gestational weight gain, and sugar-sweetened beverage consumption during pregnancy (Table 5). Children with greater birth weight had increased odds of belonging to the rapidly-rising 2L compared to the reference 3L group (OR = 1.21 95%CI 1.03, 1.43, per 100 gram increase). Additionally, children of mothers that consumed more sugar-sweetened beverages during pregnancy had increased odds of being in the steep-dropping and rebounding 1A compared to the reference 3A group (OR = 1.08 95%CI 1.01, 1.17, per 1 drink/week increase). No other significant ORs were found between the early life characteristics examined and adipokine groups.

**Table 5:** Relationships between early life factors and adipokine group membership.

			Adiponectin Group		Leptin Group		
			1A^1^	2A	1L	2L	
Early life characteristic	mean (SD)	N	OR (95% CI)	OR (95% CI)	OR (95% CI)	OR (95% CI)	
						
**Gestational age (weeks)**	38.8 (1.2)	80	1.14 (0.67, 1.96)	1.39 (0.83, 2.34)	1.48 (0.79, 2.79)	1.18 (0.66, 2.12)	
						
**Birth weight (100g)**	34.5 (4.4)	80	1.02 (0.86, 1.2)	1.01 (0.87, 1.18)	1.35 (0.88, 2.08)	1.21 (1.03, 1.43)	
						
**Birth length (cm)**	50.6 (2.5)	80	1.07 (0.82, 1.4)	1.03 (0.84, 1.27)	1.2 (0.89, 1.6)	1.21 (0.89, 1.64)	
						
**Maternal pre-pregnancy BMI (kg/m^2^)**	26.5 (4.9)	80	1.1 (0.96, 1.27)	1.08 (0.84, 1.37)	0.98 (0.84, 1.14)	1.15 (0.96, 1.38)	
						
**Maternal gestational weight gain (kg)**	14.3 (5.4)	80	0.99 (0.88, 1.13)	1.06 (0.93, 1.22)	1.08 (0.93, 1.26)	1.05 (0.9, 1.22)	
						
**Maternal SSB consumption (drinks/week)**	14.3 (10.9)	79	1.08 (1.01, 1.17)	1.04 (0.97, 1.12)	0.89 (0.77, 1.03)	0.97 (0.92, 1.03)	

OR - odds ratio, SD - standard deviation, BMI - body mass index, SSB - sugar-sweetened beverage.

1 Odds ratios are calculated in reference to group 3 of either adiponectin (3A) or leptin (3L).

## Discussion

In this study, we examined adiponectin and leptin development in Mexican American children at high risk of obesity. To our knowledge, this is the first time that adipokine trajectories have been analyzed over the entire birth to 9-year period. While leptin levels closely and positively reflected child body size at all ages, adiponectin had inverse and weaker associations with BMI at 2, 5, and 9 years. We identified three trajectory clusters for both leptin (1L, 2L, and 3L) and adiponectin (1A, 2A, and 3A). Of the perinatal factors examined, children with greater birth weight had increased odds of belonging to the rapidly-rising group 2L and those whose mothers consumed more sugar-sweetened beverages during pregnancy were at risk of being in the steep-dropping group 1A. Additionally, we found that correlations increased comparing the birth – 2, 2–5, and 5–9-year periods for both adiponectin and leptin. Taken together, our findings highlight the different roles that adiponectin and leptin have during obesity development in children and add evidence that perinatal factors may have long lasting effects on metabolic health.

Our mixture modeling analyses identified key differences in adiponectin compared to leptin development over time. For adiponectin, we observed a large decrease in levels over the 0–2-year period and suggest that this drop drives the heterogeneity in child trajectories. On the other hand, for leptin, children tended to be most separated over the 2–9-year period, with group 2L children displaying a steady rise and increased BMI and waist circumference at 9 years. We offer several reasons for these observed differences in adipokine trajectories.

In our study, leptin levels were related closely to child birth weight and BMI (Table 2) and, as a result, leptin trajectories are likely to reflect child growth. Further highlighting this, Table 4 shows that children belonging to the rapidly-rising 2L group, consistently had the highest birth weight and BMI at 2, 5, and 9 years. In fact, the leptin groups described here match closely in shape the three BMI trajectories identified by Pryor et al, who reported on low-stable, moderate and rapidly-rising BMI groups, with the latter two indistinguishable over the 5-month to 2.5-year period [2].

In contrast, although adiponectin was inversely associated with BMI at 2, 5, and 9 years, these correlations were weaker compared to those of leptin. Other factors that may affect adiponectin levels over infancy remain poorly understood. Adiponectin is known to play a critical role in insulin sensitivity, regulating glucose, triglyceride and free fatty acid metabolism [6], [34]. Given the significant differences in pre and post-partum energy regulating mechanisms, increasing energy requirements and changing body composition during infant development, adiponectin levels may more closely reflect these changes rather than of body size itself [16], [35], [36]. Further supporting this interpretation, we and others have previously shown that adiponectin is significantly related to triglyceride and lipoprotein levels, independently of BMI in 9-year-old children [19], [37].

We did observe that group 1A had the steepest drop in adiponectin levels and a slight rebound to higher values. Further, children whose mothers had higher sugar-sweetened beverage consumption had higher odds of belonging to this group. Mechanisms responsible for this association remain unclear but greater sugar-sweetened beverage consumption during pregnancy may lead to increased glucose transfer to the fetus, resulting in insulin-mediated effects on offspring adiponectin levels [38], [39]. Nevertheless, the significance of this association should be interpreted with caution, given that our data on maternal sugar-sweetened beverage consumption were questionnaire-based and may be subject to recall bias.

Importantly, it remains unclear whether there are long-term health consequences for children in group 1A. We had lipid profile data only on a small subset (N = 35) of the 80 children in this study and our data showed that group 1A children tended to have higher mean triglycerides (123.2 vs. 87.7mg/dL; N = 9 and 12 respectively), very low-density lipoproteins (24.7 vs. 17.6 mg/dL) and lower high-density lipoproteins (46.4 vs. 55.1 mg/dL) compared to group 3A. Despite the limited sample size, we suggest that children with rapid adiponectin decreases over infancy may develop adverse lipid levels and emphasize the need to further examine possible metabolic consequences of early life changes in adiponectin.

Additionally, we did observe that children with greater birth weight had increased odds of being in the rapidly-rising 2L group. This result is in line with several reports showing an association between increased birth weight and membership to more rapidly-rising BMI groups [1], [3]. Previous studies have shown that children born at either end of the birth weight spectrum are at higher risk of obesity and metabolic disorders in adulthood [40]–[43]. In this cohort, the majority of children were appropriate for gestational age (N = 70) and our results further emphasize the important role of birth weight in determining later life risk of obesity.

We also report that correlations increased comparing the birth – 2, 2–5, and 5–9-year periods for both adiponectin and leptin. Only one study has previously examined these relationships for adipokines, reporting no association for adiponectin and finding a significant and positive association for leptin, comparing child levels at birth and 3 years [17]. Our results are in agreement with those from several other reports that focused on BMI, showing that child BMI predicts adult obesity and this association strengthens with child age [44], [45]. Taken together, these data may suggest greater plasticity during the early infancy period and progressive constrainment to specific metabolic health with age.

Results of this study should be interpreted taking into account several limitations. Our analyses were performed on a relatively modest sample size of 80 children with complete adipokine data at birth, 2, 5, and 9 years. Importantly, other than BMI and waist circumference at 9 years, we had only limited data on children’s lipid profile and no other measures of metabolic health. As a result, consequences of the differing adiponectin trajectories remain unclear. Additionally, we did not explicitly include onset of puberty in our analyses. This may be an important factor to consider given that both adiponectin and leptin are suppressed by increased androgen release during puberty [46], [47]. However, data for our cohort show that child adiponectin, leptin and BMI did not yet differ by sex at 9 years (Table S1). Finally, there may be fluctuations in adipokine levels between the 0, 2, 5, and 9-year time points not assessed by our analyses.

Strengths of this study include its extensive data collection, with availability of biological samples, anthropometric measures and questionnaire-based health assessments at a variety of time points. Further, examining adipokine trajectories is particularly relevant to the CHAMACOS population, given its high prevalence of child obesity. It is also important to mention that the novel mixture-modeling approach applied here helps improve our understanding of adipokine development over childhood and obesity etiology in general.

In conclusion, our results support the idea that children may be on particular metabolic health trajectories from birth that become progressively more defined with time. We identified several potential risk factors for altered childhood adipokine levels, including increased maternal sugar-sweetened beverage consumption during pregnancy and increased child birth weight. Additionally, our longitudinal data on adiponectin and leptin highlight their different utilities, arguing that leptin remains tightly linked to body size while adiponectin is more reflective of underlying metabolic health during childhood.

Key future directions involve further exploring relationships between the *in utero* environment and adipokine trajectories. Additionally, while emerging data identify features of BMI growth curves, such as increased BMI at infancy peak and early adiposity rebound, as risk factors for later life obesity, the accompanying changes in adipokine levels remain poorly understood [5]. Finally, more research is needed to determine whether adipokine trajectories over childhood can predict adolescent and adult metabolic health.

## Supporting Information

Table S1 **Adiponectin (µg/ml), log(leptin) and child size by age and gender (N = 80).** A - adiponectin, L- log_10_(leptin), BMI - body mass index. A_0_,L_0_ - at birth, A_2_,L_2_, BMI_2_ - at 2 years, A_5_,L_5_,BMI_5_ - at 5 years, A_9_,L_9_,BMI_9_ - at 9 years. ^1^P-value refers to t-test for differences by sex within a given time point.
(DOC)
