# Supplementary material for: Adiponectin and Leptin Trajectories in Mexican-American Children from Birth to 9 Years of Age
Source: PLoS One. 2013 Oct 30;8(10):e77964. doi: 10.1371/journal.pone.0077964 (PMC3813557; doi:10.1371/journal.pone.0077964)
Supplement: Table S1 — Adiponectin (µg/ml), log(leptin) and child size by age and gender (N = 80). A - adiponectin, L- log10(leptin), BMI - body mass index. A0,L0 - at birth, A2,L2, BMI2 - at 2 years, A5,L5,BMI5 - at 5 years, A9,L9,BMI9 - at 9 years. 1P-value refers to t-test for differences by sex within a given time point. (DOC) [file pone.0077964.s001.doc]

| **Table S1: Adiponectin (μg/ml), log(leptin) and child size by age and gender (N=80) .** | | | | | | | | | | |  |  |
| --- | --- | --- | --- | --- | --- | --- | --- | --- | --- | --- | --- | --- |
|  |  | **Birth** |  |  | **2 years** |  |  | **5 Years** |  |  | **9 years** |  |
|  | **N** | **mean (SD)** | **P^1^** |  | **mean (SD)** | **P** |  | **mean (SD)** | **P** |  | **mean (SD)** | **P** |
|  |  |  |  |  |  |  |  |  |  |  |  |  |
|  |  | A_0_ (µg/mL) |  |  | A_2_ (µg/mL) |  |  | A_5_ (µg/mL) |  |  | A_9_ (µg/mL) |  |
| **Boy** | 39 | 108.4 (33.1) |  |  | 52.8 (19.4) |  |  | 38.6 (16.0) |  |  | 41.6 (21.0) |  |
| **Girl** | 41 | 117.0 (36.8) | 0.28 |  | 49.9 (21.6) | 0.53 |  | 45.1 (20.2) | 0.12 |  | 43.4 (18.4) | 0.69 |
|  |  |  |  |  |  |  |  |  |  |  |  |  |
| **All** | 80 | 112.8 (35.1) |  |  | 51.3 (20.4) |  |  | 41.9 (18.5) |  |  | 42.5 (19.6) |  |
|  |  |  |  |  |  |  |  |  |  |  |  |  |
|  |  | L_0_ |  |  | L_2_ |  |  | L_5_ |  |  | L_9_ |  |
| **Boy** | 39 | 0.92 (0.29) |  |  | 0.39 (0.14) |  |  | 0.43 (0.18) |  |  | 0.80 (0.41) |  |
| **Girl** | 41 | 1.23 (0.37) | <0.001 |  | 0.45 (0.18) | 0.12 |  | 0.55 (0.25) | 0.01 |  | 0.97 (0.50) | 0.11 |
|  |  |  |  |  |  |  |  |  |  |  |  |  |
| **All** | 80 | 1.08 (0.37) |  |  | 0.42 (0.16) |  |  | 0.49 (0.23) |  |  | 0.89 (0.47) |  |
|  |  |  |  |  |  |  |  |  |  |  |  |  |
|  |  | Birth weight (kg) |  |  | BMI_2_ (kg/m^2^) |  |  | BMI_5_ (kg/m2) |  |  | BMI_9_ (kg/m2) |  |
| **Boy** | 39 | 3.44 (0.41) |  |  | 16.9 (1.7) |  |  | 16.8 (1.82) |  |  | 20.2 (3.8) |  |
| **Girl** | 41 | 3.46 (0.47) | 0.84 |  | 17.5 (1.64) | 0.09 |  | 17.6 (2.55) | 0.1 |  | 20.4 (4.29) | 0.81 |
|  |  |  |  |  |  |  |  |  |  |  |  |  |
| **All** | 80 | 3.45 (0.44) |  |  | 17.2 (1.7) |  |  | 17.2 (2.3) |  |  | 20.3 (4.0) |  |
| A - adiponectin, L- log_10_(leptin), BMI - body mass index. | | | | | |  |  |  |  |  |  |  |
| A_0_,L_0_ - at birth, A_2_,L_2_, BMI_2_ - at 2 years, A_5_,L_5_,BMI_5_ - at 5 years, A_9_,L_9_,BMI_9_ - at 9 years. | | | | | | | | | |  |  |  |
| ^1^P-value refers to t-test for differences by sex within a given time point. | | | | | | | | |  |  |  |  |
